# HPV DNA testing improves CIN2+ risk stratification and detection of CIN2+ in delayed triage of ASCUS and LSIL. A population-based follow-up study from Western Norway

**DOI:** 10.1002/cam4.171

**Published:** 2013-12-17

**Authors:** Elisabeth B Budal, Hans K Haugland, Robert Skar, Bjørn O Mæhle, Tone Bjørge, Olav K Vintermyr

**Affiliations:** 1 Department of Pathology, Haukeland University HospitalN-2021, Bergen, Norway; 2 Section for Pathology, Department of Clinical Medicine, University of BergenN-5021, Bergen, Norway; 3 Department of Global Public Health and Primary Care, University of BergenBergen, Norway; 4 Norwegian Institute of Public HealthBergen, Norway

**Keywords:** ASCUS, cervical cancer, delayed triage, HPV, LSIL

## Abstract

In Norway, Pap smears with atypical squamous cells of uncertain significance (ASCUS) and low-grade squamous intraepithelial lesions (LSIL) are triaged after 6 months. The aim of the study was to evaluate effects of implementing human papillomavirus (HPV) test (2005) in delayed triage of ASCUS and LSIL in a cohort of women from Western Norway. After a survey of 119,469 cervical Pap smears during 2005–2007, a total of 1055 women with an index ASCUS or LSIL were included in the study and followed up for 3–6 years with respect to progression into cervical intraepithelial neoplasia grade 2 or worse (CIN2+). Overall sensitivity for detection of CIN2+ with HPV testing and cytology was 96% and 72%, respectively. The sensitivity for detection of CIN2+ was not affected by age, but the specificity of the HPV test increased with age. Thus, for the age groups <34 years, 34–50 years, and >50 years, the specificity of a positive HPV test to detect CIN2+ was 47%, 71%, and 82%, respectively. Positive predictive values for CIN2+ in women with positive cytology, positive HPV test, negative cytology, negative HPV test, or negative HPV and cytology tests were 52%, 41%, 8%, 1.5%, and 0.4%, respectively. HPV testing resulted in a net 22% increased detection of CIN2+. Fifty-six percent of CIN2+ was detected at an earlier time point with HPV testing in triage. Implementation of HPV testing in delayed triage of ASCUS and LSIL improved the stratification of CIN2+ risk and increased CIN2+ detection and at an earlier time point than with triage by cytology alone.

## Introduction

Uterine cervical cancer is the second most common cancer among women worldwide 1. Squamous cell carcinomas account for about 75–80% of the new cancer cases 2. They develop through a multistep sequence of events from low- through high-grade cellular lesions and then into cancer 3,4. The cause of the disease is closely associated with genital high-risk human papillomavirus (hrHPV) infection 5–7, normally starting in the transitional zone between the squamous and the columnar epithelium 8.

Most of the hrHPV infections regress spontaneously, whereas some progress to high-grade lesions and eventually into cancer 9. It is generally assumed that it will take more than 10 years from infection until development of cancer 10. Most developed countries have established nationwide screening programs to detect and treat high-grade cellular lesions and to monitor atypical squamous cells of uncertain significance (ASCUS) and low-grade squamous intraepithelial lesions (LSIL). These programs are mainly based on cytology as the primary screening method. The approach has proven useful in the sense that the incidence rates of cervical cancer have decreased 11–13 despite an increasing prevalence of genital hrHPV infections in Western countries 14.

A hrHPV infection can be detected through HPV testing. This method is more sensitive, but less specific than cytology to detect clinical relevant infections 15,16. Due to the higher sensitivity, some countries have in part implemented HPV testing in primary screening 17. Recent randomized trials have shown a higher detection of CIN2 or worse (CIN2+) using HPV testing in primary screening as compared to cytology 18–22. Studies also indicate that HPV-negative women can be referred to routine screening with longer screening intervals 23,24.

In 1995, an organized screening program for cervical cancer was introduced in Norway. Women with an index ASCUS or LSIL Pap smear were triaged with a control smear after 6 months. In 2005, HPV testing was introduced in the triage of ASCUS and LSIL within the program.

In this study, we report on women with an index sample of ASCUS or LSIL and monitoring with cytology and HPV testing in delayed triage. A major aim of this study was to evaluate if HPV testing would recover more CIN2+ than cytology alone and enable a more differentiated follow-up screening algorithm in selected groups of women.

## Material and Methods

### Overall study design

In Norway, cervical screening is organized as a triennial screening to all women, aged 25–69 years, and with a written reminding letter to all non-attending women.

A total of 119,469 smears were screened during a 3-year period from 2005 to 2007, and patients with ASCUS or LSIL were identified. Among these, 1055 patients were enrolled in the study. The patients were followed up over the next 3–6 years with respect to progression into CIN2+ based on diagnostic cervical biopsies and/or cervical resected cones. The more severe lesion was recorded. All samples were examined at Haukeland University Hospital, which is a regional hospital of Western Norway.

### Definitions

*Positive cytology* means a Pap smear 25 showing ASCUS or worse (ASCUS+). *Negative cytology* means a normal or reactive smear without suspicion of ASCUS+. *Index sample* refers to the initial Pap smear that showed ASCUS or LSIL and led to enrollment in the study. *The first follow-up test* refers to the first control test performed after the index sample.

### Inclusion criteria

All women encountered with ASCUS or LSIL during the period 2005–2007 were evaluated for enrollment. Women with a positive cytology or positive HPV test during the previous 2 years were excluded. These women were not considered to have a de novo cytologically encountered cervical lesion. Lack of such information did, however, not exclude women from being enrolled in the study, as many of these women had never attended a screening program previously. Furthermore, the HPV test should be sampled together with the first follow-up smear or less than 1 month before or after the smear was taken.

### Patient material

**Figure 1:**
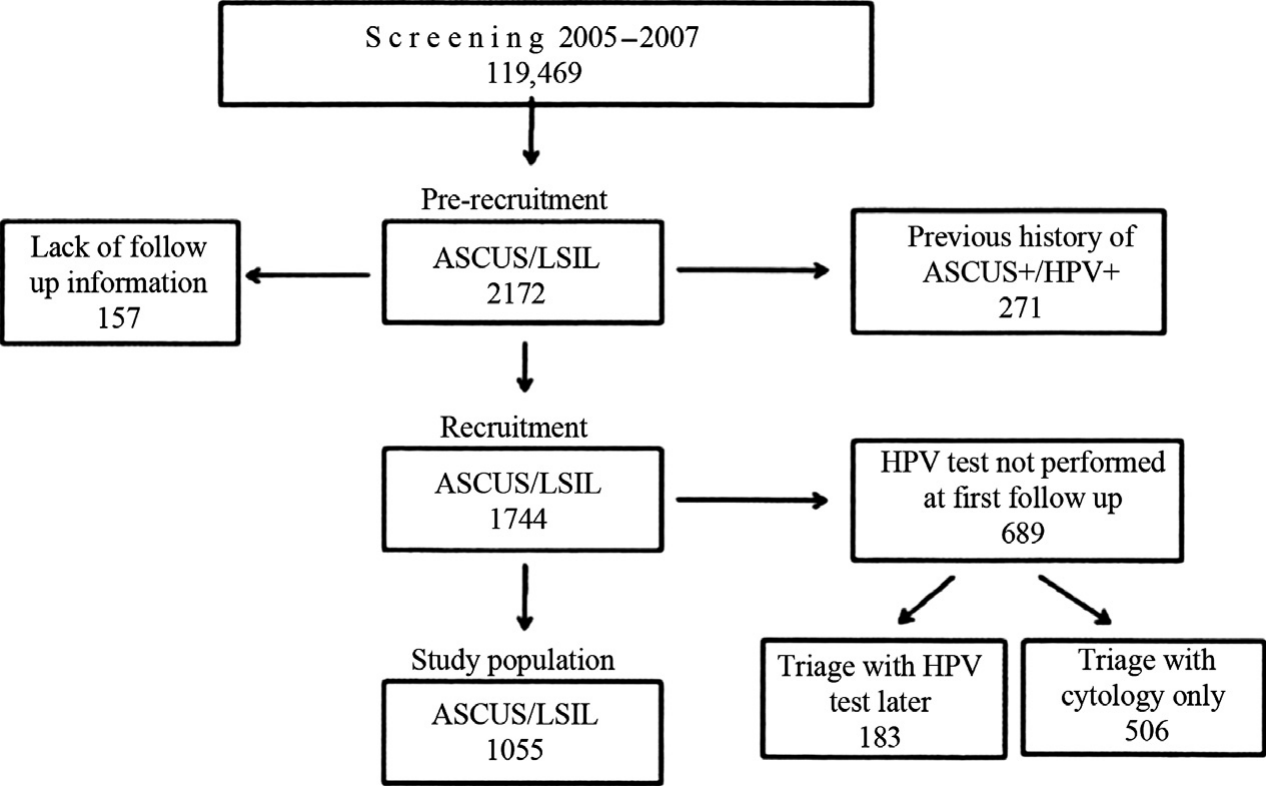
Flowchart of study inclusion.

A total of 2172 women with ASCUS or LSIL were identified. Women with (1) prior abnormal smear(s) and/or positive HPV tests, (2) lack of follow-up information, and incomplete HPV follow-up of ASCUS/LSIL were omitted from the study (Fig. 1). The study has been approved by the Regional Committee for Ethics in Research (2013/805).

### HPV DNA test

A separate brush sample for HPV testing was normally taken in parallel with the Pap smear. The HPV samples were stored at 4°C until further processing. The HPV DNA test (*digene* HC2 HPV DNA test, QIAGEN, Hombrechtikon, Switzerland) identifies the presence of hrHPV types 16, 18, 31, 33, 35, 39, 45, 51, 52, 56, 58, 59, and 68 without notifying the presence of any single hrHPV subtype. The HPV test results were normally not available to the cytotechnicians screening the smears, but available to the pathologist who finally signed out the smears.

### Effect of HPV test in delayed triage versus cytology alone

In delayed triage of ASCUS and LSIL, standard follow-up is with repeat cytology after 6 months. Since 2005, HPV testing has been used together with cytology for women aged 25–69 years. In this study, the effect of HPV testing on recovery of CIN2+ as well as the time interval from index ASCUS/LSIL until detection of CIN2+ was evaluated. For this purpose, all cytology and surgical pathology reports of all the women included in the study were examined.

A major difference in the triage algorithm with HPV testing (and cytology) versus cytology alone is that HPV-positive women will be referred for immediate colposcopy if cytology is positive. HPV-positive women with negative cytology are also followed up with new cytology smears and/or colposcopy until the HPV test is negative (regression of disease) or has progressed to CIN2+. All HPV-negative women are referred back to routine screening. In the triage modality with cytology alone, an index ASCUS sample is referred back to routine screening if the follow-up cytology is normal (negative). For index LSIL, two ensuing cytology smears must be normal before the woman is referred back to routine screening. A woman will be referred for colposcopy if two ensuing repeat control tests show persistent equivocal or low-grade lesions. In both screening modalities, immediate colposcopy will follow if cytology shows high-grade lesion or suspicion of its presence.

An evaluation of the cervical test records in relation to the clinical management algorithms was done by three cytopathologists (E. B. B., H. K. H., O. K. V.). A complete outline of the management guidelines of ASCUS/LSIL is available at The Cancer Registry of Norway (http://www.kreftregisteret.no).

### Statistical analysis

Assessment of sensitivity, specificity, and positive predictive values (PPVs) for detection of CIN2+ in cytology and with HPV testing refer to the first follow-up cytology and HPV test taken after index ASCUS/LSIL. The cytology test was considered correctly positive if ASCUS+ identified a woman with CIN2+ or one who developed CIN2+ during follow-up. This definition of true positivity in cytology has also been used by others 26. Similarly, the HPV test was considered correctly positive if the woman had CIN2+ or developed CIN2+ during follow-up.

Cumulative incidence rates were calculated using the method described by Kaplan and Meier 27. Pearson's chi-squared test was used to evaluate the associations between categorical variables for results obtained in cytology versus HPV testing.

## Results

### General observations

Sixty-eight percent of the women in the study group (Fig. 1) had their first follow-up (control) test 4–12 months after the index sample, 19% earlier than 4 months, and 12% later than 12 months. At the first control of ASCUS/LSIL, 515 women had negative cytology and negative HPV test, 260 positive cytology and positive HPV test, 246 negative cytology and positive HPV test, and 34 had positive cytology and negative HPV test. The HPV test was positive in 37% of women with index ASCUS and in 70% of women with index LSIL, respectively (Table 1).

**Table 1:** Number (No) of women in various age groups with respect to (1) ASCUS and LSIL (index smear [cytology]), (2) test results at the first control, (3) HPV test results at first control versus index smear, and (4) the whole study population (all women).

	No of women by age at baseline			
Age groups	<34 years No (%)	34–50 years No (%)	>50 years No (%)	All ages
Index smear (cytology)				
ASCUS	**217** *(31)*	**320** *(46)*	**161** *(23)*	**698**
LSIL	**209** *(59)*	**122** *(34)*	**26** *(7)*	**357**
Test results 1st control				
Cyt. pos.	**135** *(46)*	**124** *(42)*	**35** *(12)*	**294**
Cyt. neg.	**289** *(38)*	**320** *(42)*	**152** *(20)*	**761**
HPV pos.	**275** *(54)*	**190** *(38)*	**41** *(8)*	**506**
HPV neg.	**151** *(28)*	**252** *(46)*	**146** *(27)*	**549**
HPV test 1st control/index cytology				
HPV pos./ASCUS index	**123** *(48)*	**103** *(40)*	**30** *(12)*	**256**
HPV neg./ASCUS index	**94** *(21)*	***217** (49)*	**131** *(30)*	**442**
HPV pos./LSIL index	**152** *(61)*	**87** *(35)*	**11** *(4)*	**250**
HPV neg./LSIL index	**57** *(53)*	**35** *(33)*	**15** *(14)*	**107**
All tests (all women)	426 (40)	442 (42)	187 (18)	1055

HPV, human papillomavirus; LSIL, low-grade squamous intraepithelial lesions.

Number of women given in bold, fraction (%) of women given in italic.

### Age groups in relation to ASCUS, LSIL, HPV, and CIN2+ detection

Most women with LSIL were younger than 34 years, whereas most women with ASCUS were older than 34 years (Table 1). A skewed age distribution of ASCUS and LSIL was similarly reflected in a high prevalence of HPV positivity in young (<34 years) as compared to elderly women (>50 years). Thus, for the age groups <34 years, 34–50 years, and >50 years, the ASCUS/LSIL ratio was 1.0, 2.6, and 6.2. HPV positivity in the LSIL group was 73%, 71%, and 57%, and HPV positivity in the ASCUS group was 57%, 32%, and 19%, respectively.

The significance of cytology and HPV testing for detection of women with CIN2+ was then evaluated in delayed triage. Among 1055 women with index ASCUS or LSIL, 214 CIN2+ were recovered during the observation period of 3–6 years (Table 2). The sensitivity for detection of CIN2+ with HPV testing was 96% and for cytology 72%. The sensitivity for detection of CIN2 in the age groups <34 years, 34–50 years, and >50 years was 70%, 73%, and 75% in cytology and 97%, 97%, and 83% with HPV testing, respectively. The specificity for detection of CIN2+ in the age groups <34 years, 34–50 years, and >50 years was 81%, 84%, and 85% in cytology and 47%, 71%, and 82% with HPV testing, respectively. Thus, the specificity of cytology to identify CIN2+ was unaffected by age, whereas the specificity of HPV test to identify CIN2+ was affected by age. Ninety-four percent of all CIN2+ were observed in the age groups below <50 years and more than half (51%) of all CIN2+ were recovered in women <34 years (Table 2).

**Table 2:** Number (No) of women in various age groups developing CIN2+ during 3–6 years of follow-up after index ASCUS or LSIL in relation to the whole study population (All tests, lower lane) and in relation to observed findings in cytology and/or HPV test at their first follow-up control.

	Recovery of CIN2+ by age at baseline			
Age groups	<34 years No (%)	34–50 years No (%)	>50 years No (%)	All ages
Test results 1st control				
HPV pos.	**106** *(51)*	**90** *(44)*	**10** *(5)*	**206**
Cyt. pos.	**76** *(50)*	**68** *(44)*	**9** *(6)*	**153**
HPV pos./Cyt. pos.	**74** *(50)*	**66** *(45)*	**7** *(5)*	**147**
Cyt. neg	**33** *(54)*	**25** *(41)*	**3** *(5)*	**61**
HPV neg.	**3** *(38)*	**3** *(38)*	**2** *(25)*	**8**
HPV neg/Cyt. pos	**2** *(33)*	**2** *(33)*	**2** *(33)*	**6**
HPV neg. Cyt. neg.	**1** *(50)*	**1** *(50)*	**0** *(0)*	**2**
All tests (all woman)	**109** *(51)*	**93** *(44)*	**12** *(6)*	**214**

HPV, human papillomavirus; LSIL, low-grade squamous intraepithelial lesions.

Number of women given in bold, fraction (%) of women given in italic.

### Risk stratification of women with HPV test in triage of ASCUS/LSIL

**Figure 2:**
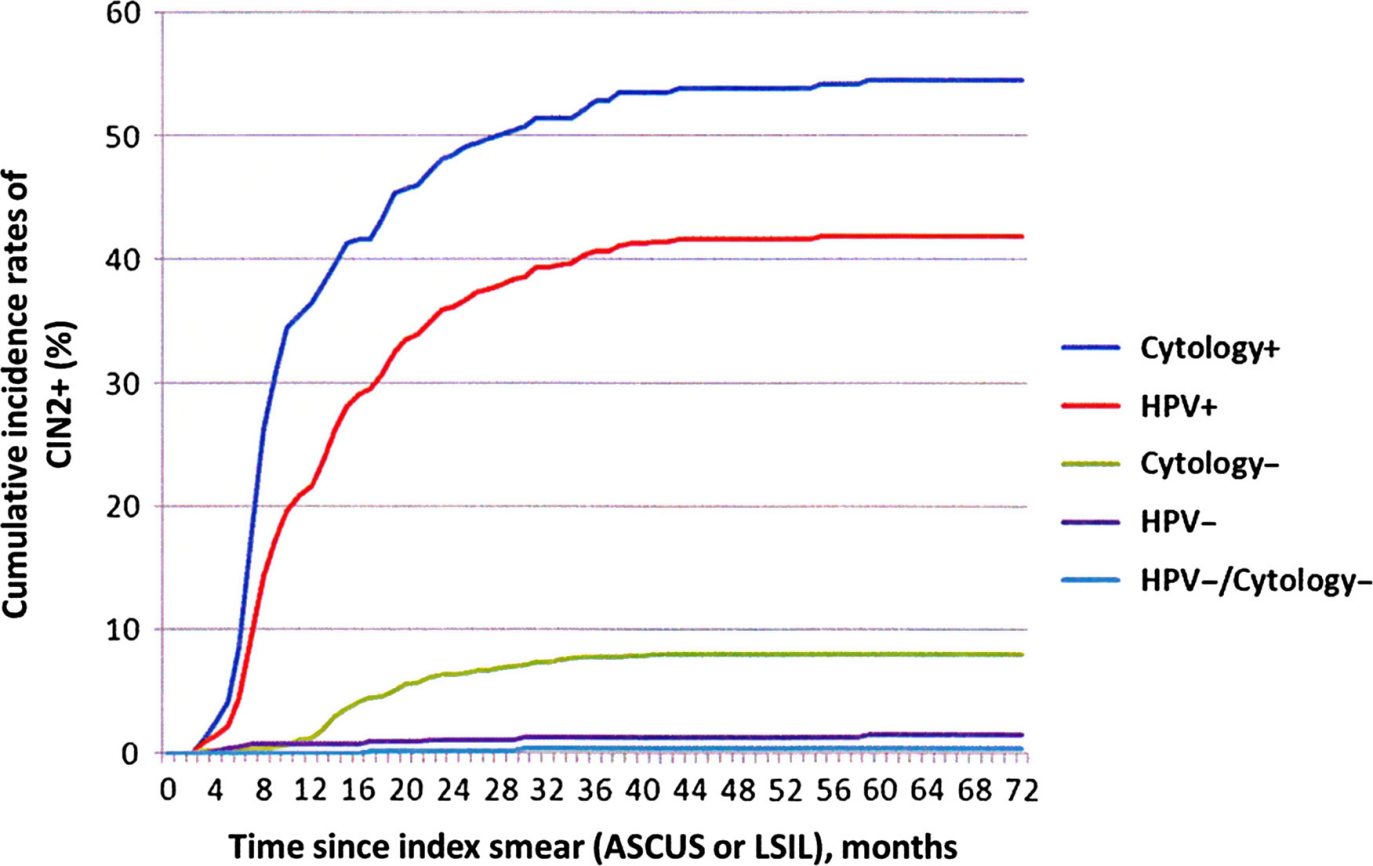
Cumulative incidence rates for CIN2+ based on test (HPV and cytology) results at the first follow-up control in women with an index ASCUS or LSIL (Kaplan–Meyer plots). HPV, human papillomavirus; LSIL, low-grade squamous intraepithelial lesions.

Cumulative incidence rates of CIN2+ in women with positive cytology, positive HPV test, negative cytology, or negative HPV test were determined. The risk of CIN2+ was high during the first 2 years after index ASCUS or LSIL, and then no further increase was seen (Fig. 2). A negative HPV test was superior to a negative cytology test to predict a low risk for CIN2+.

**Figure 3:**
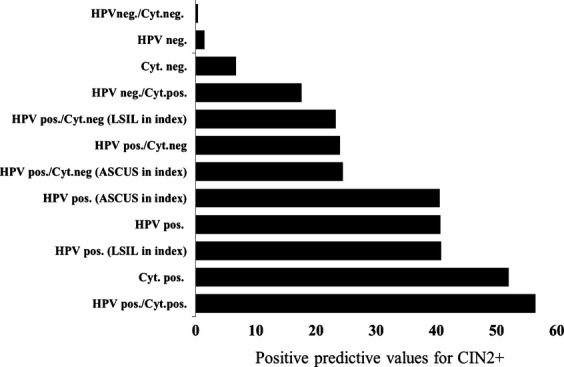
Positive predictive values for CIN2+ based on test (HPV and cytology) results at the first follow-up control in women with an index ASCUS or LSIL. HPV, human papillomavirus; LSIL, low-grade squamous intraepithelial lesions.

The risks of CIN2+ in relation to possible test results were further calculated and reported as PPVs for CIN2+ (Fig. 3). PPVs for CIN2+ given a positive HPV test were high in this study, indicating a strong selection of clinical relevant HPV-positive lesions by the used modality for triaging of ASCUS or LSIL. The risks of CIN2+ in the ASCUS and LSIL group of women were closely similar if the HPV tests were positive (Fig. 3). The results of the HPV testing also markedly affected the risk of CIN2+ in cytology. Thus, the risk (PPV) of CIN2+ in women with negative cytology was 6.7% as compared to 0.4% if the HPV test was negative (Fig. 3).

### Effect of HPV testing on CIN2+ detection

**Figure 4:**
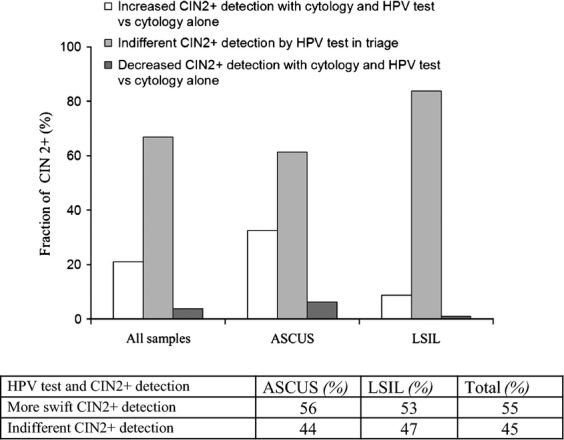
Effect of cytology and HPV test versus cytology alone on detection of CIN2+ (upper panel) and on the time course for detection of CIN2+ (lower panel) in triage of ASCUS and LSIL. HPV, human papillomavirus; LSIL, low-grade squamous intraepithelial lesions.

A delayed triage algorithm with cytology and HPV testing versus cytology alone was evaluated for recovery of CIN2+ in women with index ASCUS or LSIL. A net 22% increased CIN2+ detection (*P* = 0.009) was observed by HPV testing in triage as compared to cytology alone (Fig. 4, upper panel). This effect was most prominent in women with index ASCUS. In this group, HPV testing led to a net 39% increase in CIN2+ recovery as compared to cytology alone (*P* = 0.004). In index LSIL women, HPV testing led to only a moderate 8.5% increased detection of CIN II (*P* = 0.4).

Then, the two various triage modalities with cytology and HPV test combined versus cytology alone were evaluated with respect to the time delay from index ASCUS/LSIL until detection of CIN2+. For that purpose, all the cytology and surgical pathology reports of women with CIN2+ were reviewed (see Methods section). In the triage modality with cytology and HPV testing CIN2+ was detected at an earlier time point than in the triage modality with cytology alone (Fig. 4, lower panel). Thus, with index ASCUS or LSIL Pap smear CIN2+ was detected at an earlier time point in 56% and 53% of the cases, respectively.

## Discussion

In this study, we addressed the effects of implementing HPV testing in delayed triage of ASCUS and LSIL in the organized population-based cervical cancer screening program in Norway. It was noted that the significance of ASCUS in terms of the risk for CIN2+ was similar to LSIL when corrected for HPV status. High PPVs of HPV-positive women for CIN2+ suggested that the clinically relevant HPV infections were identified by the delayed triage algorithm. HPV testing not only led to an increased CIN2+ detection but also to a shorter time interval until CIN2+ was detected.

Apart from some women who were excluded due to a previous history of ASCUS+ or lack of clinical follow-up information, 689 women (39%) with ASCUS or LSIL at baseline did not have a HPV test at their first follow-up smear (Fig. 1). In the study (HPV) group, CIN2+ was recovered in 20% of the women, whereas in the group of 689 non-HPV tested women 19%, CIN2+ was detected (see Fig. 1). This indicated that there was no major difference in CIN2+ prevalence between these two groups.

In the ALT's study, triage of ASCUS with HPV test was clinically useful, but of limited value in triage of LSIL as most (82.9%) of these women were reported to be HPV positive 28. In this study, using the modality of delayed triage, HPV test was positive in 37% of women with ASCUS and in 70% of women with LSIL (Table 1). This indicated a more clinically relevant role of HPV testing in delayed triage of LSIL. Moreover, in women >50 years, 86% of ASCUS/LSIL smears were of the ASCUS type and only 19% of the latter were positive for HPV. The same age-dependent effect was observed in the ALT's study and also more recently by Gyllensten and coworkers 29.

The cumulative incidence rates of CIN2+ in the HPV-positive women were high in this study (Fig. 2) as compared to studies where HPV testing was done at baseline (reflex HPV testing) 30–32. A higher cumulative incidence rate of CIN2+ has been reported in women with persistent HPV infection 33,34. In a recent study, two thirds of all HPV infections underwent spontaneous regression within 1 year 35. It has been reported that a less aggressive approach of HPV testing at baseline allow more HPV infections to undergo spontaneous regression before being triaged 36. The high PPVs for CIN2+ in HPV-positive women in this study also indicated that clinically relevant lesions were found using a strategy with delayed HPV triage of ASCUS and LSIL.

HPV testing has for a long time been shown to have a superior negative predictive value (NPV) for developing CIN2+ at baseline 23,37–39. Similar results were also observed in this study where baseline ASCUS and LSIL smears were triaged 6–12 month later with cytology and HPV testing (Fig. 3). Although few in number, women with negative HPV test and positive cytology had a considerably increased risk of CIN2+ (Fig. 3). This should raise some concern as to rely too heavily on a single negative HPV test, if the cytology is positive.

In this study, an effect of cytology and HPV testing versus cytology alone on detection of CIN2+ in triage of ASCUS and LSIL was based on the evaluation of all cytology and surgical pathology reports of each woman with CIN2+ included in the study. This approach will only give estimates for detection of CIN2+ with and without HPV testing in triage of ASCUS and LSIL, and is related to the assumption that women triaged with cytology alone fully adopt the clinical management algorithm. Also, the effect of implementing HPV testing in triage is based on results at the first follow-up after index ASCUS and LSIL. This means that the potential for a secondary catch up of a previously missed CIN2+ in the first screening round with cytology or HPV test is not accounted for by this approach.

We report an estimated net 22% increased recovery of CIN2+ by the use of HPV testing versus cytology alone (Fig. 4). More than half of the CIN2+ lesions were detected at an earlier time point with HPV testing in triage as compared to cytology alone (Fig. 4, lower panel). However, this could represent a caveat in the sense that not only the numbers of CIN2+ lesions detected but also their potential to regress and progress into cancer are of importance. Thus, a more protracted detection of CIN2+ might identify fewer high-grade lesions, but of higher clinical relevance 21. An increased detection of CIN2+ has been registered after implementation of HPV testing in triage of ASCUS/LSIL in Norway 40. Randomized controlled trial(s) on the effect of HPV testing in triage of ASCUS/LSIL have not been performed in Norway.

It was observed that in a screening modality based on delayed triage of ASCUS/LSIL very few women with an index ASCUS smear above 50 years of age were HPV-positive, and for those the specificity of a positive HPV test for CIN2+ approached the specificity of a positive cytology test. Furthermore, HPV testing led to an increased and more rapid detection of CIN2+ and to an improved stratification of CIN2+ risk. Thus, positive HPV tests showed high PPVs for CIN2+, indicating that the clinical relevant lesions were identified in this screening algorithm. A particular low risk for CIN2+ was observed in HPV test negative women indicating that a more differentiated routine screening protocol could be advocated for these women.

## Conflict of Interest

None declared.
